# MRP3 as a novel resistance factor for sorafenib in hepatocellular carcinoma

**DOI:** 10.18632/oncotarget.6889

**Published:** 2016-01-12

**Authors:** Tetsu Tomonari, Shunsaku Takeishi, Tatsuya Taniguchi, Takahiro Tanaka, Hironori Tanaka, Shota Fujimoto, Tetsuo Kimura, Koichi Okamoto, Hiroshi Miyamoto, Naoki Muguruma, Tetsuji Takayama

**Affiliations:** ^1^ Department of Gastroenterology and Oncology, Institute of Biomedical Sciences, Tokushima University Graduate School, Tokushima City, 770-8503, Japan

**Keywords:** hepatocellular carcinoma, sorafenib, resistance, transporter, MRP3

## Abstract

The mechanism of resistance of hepatocellular carcinoma (HCC) to sorafenib is unknown and no useful predictive biomarker for sorafenib treatment has been reported. Accordingly, we established sorafenib-resistant HCC cells and investigated the underlying mechanism of resistance to sorafenib. Sorafenib-resistant cell lines were established from the HCC cell line PLC/PRF5 by cultivation under continuous exposure to increasing concentration of sorafenib. The IC50 values of the 2 resistant clones PLC/PRF5-R1 and PLC-PRF5-R2 were 9.2±0.47 μM (1.8-fold) and 25±5.1 μM (4.6-fold) respectively, which were significantly higher than that of parental PLC/PRF5 cells (5.4±0.17 μM) (*p* < 0.01 respectively), as determined by MTT assay. Western blot analysis of signal transduction-related proteins showed no significant differences in expression of AKT/pAKT, mTOR/pmTOR, or ERK/pERK between the 2 resistant clones versus parent cells, suggesting no activation of an alternative signal transduction pathway. Likewise, when expression of membrane transporter proteins was determined, there were no significant differences in expression levels of BSEP, MDR1, MRP2, BCRP, MRP4 and OCT1 between resistant clones and parent cells. However, the expression levels of MRP3 in the 2 resistant clones were significantly higher than that of parent cells. When MRP3 gene was knocked down by siRNA in PLC-PRF5-R2 cells, the sensitivity of the cells to sorafenib was restored. In the analysis of gene mutation, there was no mutation in the activation segment of Raf1 kinase in the resistant clones. Our data clearly demonstrate that the efflux transporter MRP3 plays an important role in resistance to sorafenib in HCC cells.

## INTRODUCTION

Hepatocellular carcinoma (HCC) is the third-leading cause of cancer-related death worldwide [1]. Most patients with HCC are diagnosed at an advanced stage, when curative therapies, such as surgical resection and percutaneous ablation, are of limited utility. Since a majority of HCC present intrinsic resistance to many cytotoxic anti-cancer agents, interventional treatment such as transarterial embolization had been applied for advanced HCC [2, 3]. Recently, it has been reported that sorafenib, a multitargeted tyrosine kinase inhibitor that blocks Raf kinase, platelet-derived growth factor receptor (PDGFR) and vascular endothelial growth factor receptors (VEGFR), significantly improved overall survival in patients with advanced HCC in large-scaled multicenter phase III trials [4, 5]. Therefore, systemic treatment with sorafenib is currently recommended for advanced stage HCC in the treatment algorithm of HCC worldwide [6]. However, the response rate for sorafenib in HCC is very low (i.e., 2 - 3%) [4, 5].

Abou-Alfa and associates investigated pERK expression in HCC tissues by immunohistochemistry, and demonstrated that pERK expression is closely associated with the effect of sorafenib [7]. This is reasonable because sorafenib blocks Raf kinase thereby inhibiting the downstream MEK/ERK pathway. However, contradictory results have been reported. Patients with high expression of pERK, demonstrated by immunohistochemistry, showed significantly shorter overall survivalthan those with low expression of pERK [8, 9]. Therefore, it remains unclear whether pERK is a predictor of the therapeutic response to sorafenib in HCC. Thus, no useful predictive biomarker for the therapeutic response to sorafenib has been identified to date. Chen and associates established sorafenib-resistant cell lines from the HCC cell line Huh7 [10]. They investigated the mechanism of resistance to sorafenib in these cells, and demonstrated that the AKT/mTOR pathway was alternatively activated instead of the RAS/RAF/MEK/ERK pathway that was blocked by sorafenib in resistant clones. However, it is unknown whether or not activation of this alternative pathway is entirely responsible for sorafenib resistance.

It is well known that multidrug resistant transporter proteins such as MDR1 and MRP2 are overexpressed in many kinds of cancer cells that are resistant to anticancer drugs. Overexpression of MDR1 has beenreported to be associated with resistance to several anticancer drugs in hematological malignancies, brain tumors, lung cancers, renal cell cancers, and ovarian cancers [11–16]. Overexpression of MRP2 is also reportedly associated with resistance to anticancer drugs in lung cancers, renal cell cancers, bladder cancer and colorectal cancers [17–20]. Since there are many transporters in hepatocytes, including MDR1 and MRP2, it is plausible that theiroverexpression is involved in resistance to sorafenib. However, there have been no reports that haveinvestigated this possible relationship between sorafenib resistance and transporter expression. In addition, it has been reported that drug resistance to molecular targeting agents is sometimes induced by genetic mutation in the targeted molecules. The resistance to imatinib in gastrointestinal stromal tumors (GISTs)is acquired by a mutation in the ATP binding site of c-kit, a molecular target of imatinib [21, 22]. Similarly, the resistance to gefitinib is acquired in lung cancer by a mutation of EGFR gene, a target molecule of gefitinib [23, 24]. However, there have been no reports that have investigated mutations of Raf1 kinase, an important target molecule forsorafenib.

Therefore, in this study, we first established sorafenib-resistant cell lines, and investigated expression of AKT/pAKT and mTOR/pmTOR, key molecules in the alternative PIK3/AKT/mTOR pathway. We then investigated the expression of several important membrane transporter proteins in the resistant cells. We finally performed mutational analysis of molecular targets of sorafenib in resistant cells.

## RESULTS

### Resistance of PLC/PRF5-R1 and PLC/PRF5-R2 clones to sorafenib

**Figure 1:**
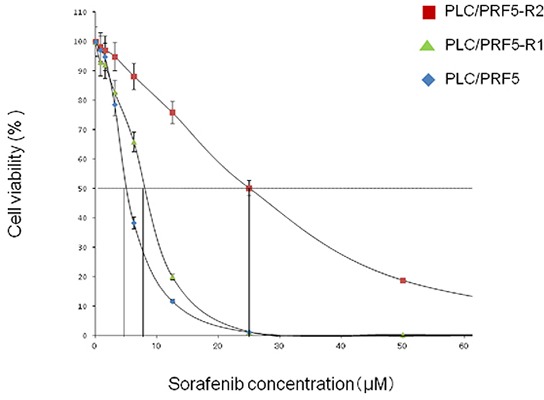
Resistance of PLC/PRF-R1 and PLC/PRF5-R2 cell lines to sorafenib The sensitivities of PLC/PRF5-R1, PLC/PRF5-R2 and PLC/PRF5 cells to sorafenib were assessed by MTT assay. The IC50 values were calculated by non-linear regression analysis.

We first established a sorafenib-resistant cell line (mixed population) from parental PLC/PRF5 cells, and isolated 2 sorafenib-resistant clones by using a limiting-dilution method as described in Materials and Methods. We then examined IC50 values of the 2 clones (PLC/PRF5-R1, PLC/PRF5-R2) by MTT assay (Figure 1). The IC50 values for PLC/PRF5-R1 and PLC-PRF5-R2 were 9.2 ± 0.47 μM (1.8-fold) and 25 ± 5.1 μM (4.6-fold) respectively, demonstrating significantly higher values than that of parental cells (5.4 ± 0.17 μM) (*p* < 0.01 and *p* < 0.01, respectively). Thus, we were able to establish sorafenib-resistant clones that showed weak or strong resistance to sorafenib.

### Expression of AKT/pAKT and mTOR/pmTOR in sorafenib-resistant clones

**Figure 2:**
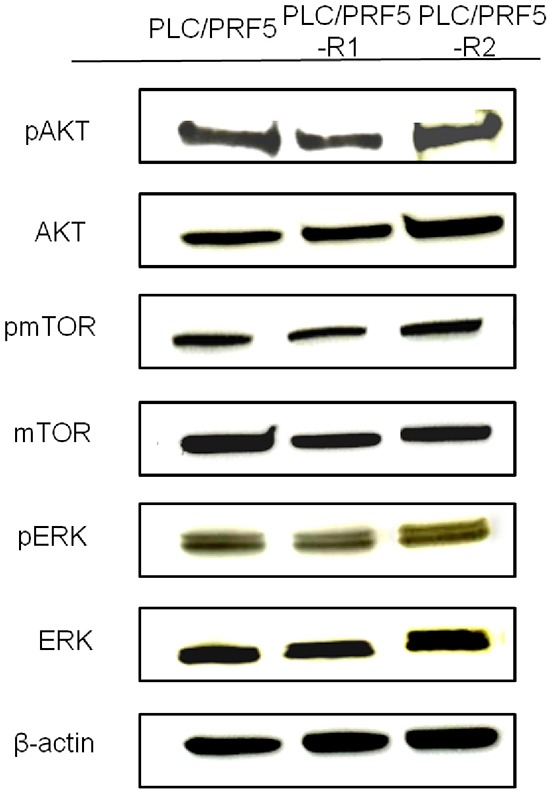
Expression of AKT/pAKT, mTOR/pmTOR, and ERK/pERK in sorafenib-resistant cells The expression of pAKT, AKT, pmTOR, mTOR, pERK, and ERK in PLC/PRF5, PLC/PRF5-R1 and PLC/PRF5-R2 cells wasexamined by Western blot analysis. β-actin was used as an internal control.

To examine if the alternative AKT/mTOR pathway is activated in the resistant clones, we investigated expression of AKT/pAKT and mTOR/pmTOR in these cells by Western blot analysis (Figure 2). However, no significant difference was observed in the bands for pAKT and AKT between resistant clones and parent cells. Likewise, no significant difference was observed in the expression of pmTOR and mTOR between resistant clones and parent cells. Thus, it became evident that the AKT/mTOR signaling pathway was not activated in our sorafenib-resistant clones. In addition, in the analysis of ERK/pERK expression, no significant difference was observed between the resistant clones and parent cells.

### Up-regulation of MRP3 in sorafenib-resistant clones

**Figure 3:**
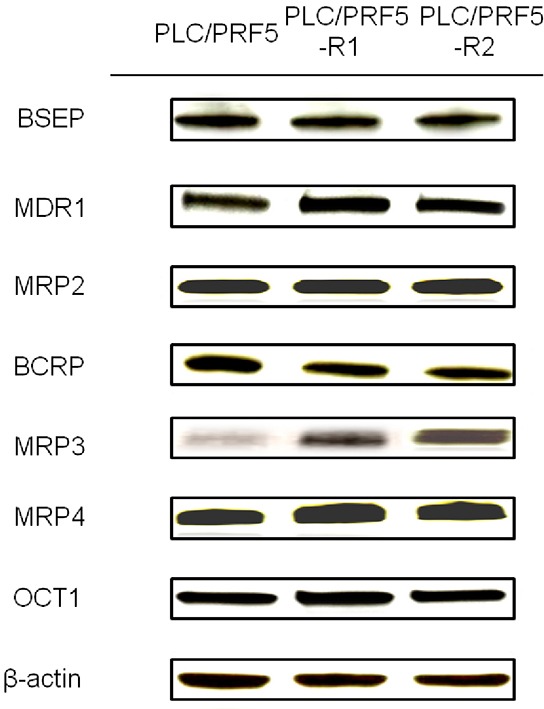
Expression of membrane transporters in sorafenib-resistant cells Expression of BSEP, MDR1, MRP2, BCRP, MRP3, MRP4, and OCT1 in PLC/PRF5, PLC/PRF5-R1 and PLC/PRF5-R2 cells wasexamined by Western blot analysis. β-actin was used as an internal control.

We investigated protein expression levels of major efflux transporters (BSEP, MDR1, MRP2, BCRP and MRP3) and influx transporters (MRP4 and OCT1) in PLC/PRF5-R1, PLC/PRF5-R2 and PLC/PRF5 cells by Western blot analysis (Figure 3). There were no significant differences in the bands for BSEP, MDR1, MRP2, BCRP, MRP4 and OCT1 among PLC/PRF5-R1, PLC/PRF5-R2 and PLC/PRF5 cells. However, the expression level of MRP3 was higher in PLC/PRF5-R1 and was even higher in PLC/PRF5-R2 cells than in PLC/PRF5 cells. Thus, the efflux transporter MRP3 was up-regulated in sorafenib-resistant clones depending on the strength of resistance, suggesting that MRP3 protein transports sorafenib outside the cells resulting in acquisition of sorafenib resistance.

### Knockdown of MRP3 restored sorafenib sensitivity

**Figure 4:**
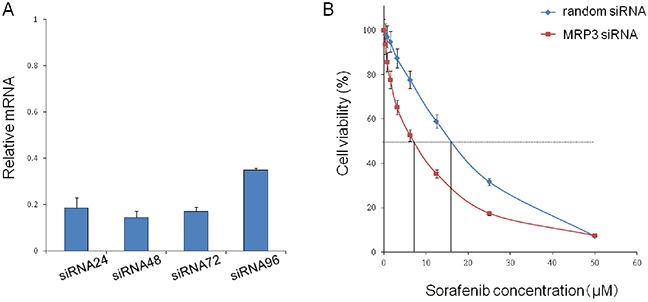
Knockdown of the MRP3 gene in PLC/PRF5-R2 cells and resulting change in sensitivity to sorafenib **A.** The MRP3 gene in PLC/PRF5-R2 cells was knocked down by siRNA. The relative mRNA levels at 24 h, 48 h, 72 h and 96 h were determined by Taqman PCR. **B.** The viabilityof PLC/PRF-R2 cells treated with siRNA or random RNA was assessed by MTT assay.

In order to prove that MRP3 is closely associated with sorafenib resistance, we knocked down theMRP3 gene in PLC/PRF5-R2 cells using siRNA and investigated the change of sensitivity to sorafenib. The relative mRNA levels of MRP3 in the knocked-down cells (PLC/PRF5-R2/si) were suppressed to 20% or less at 24 – 72 h after transfection of siRNA as compared with that of control cells (PLC/PRF5-R2/ra) (Figure 4A). The IC50 value of PLC/PRF5-R2/si cells was 7.2 ± 1.9 μM, which was significantly lower than that of control cells (15.9 ± 2.1 μM) (Figure 4B). Thus, knockdown of MRP3 in sorafenib-resistant cells restored sensitivity to sorafenib, suggesting that MRP3 plays an important role for acquisition of resistance to sorafenib.

### No mutation in the activation segment of Raf1 kinase in resistant clones

**Figure 5:**
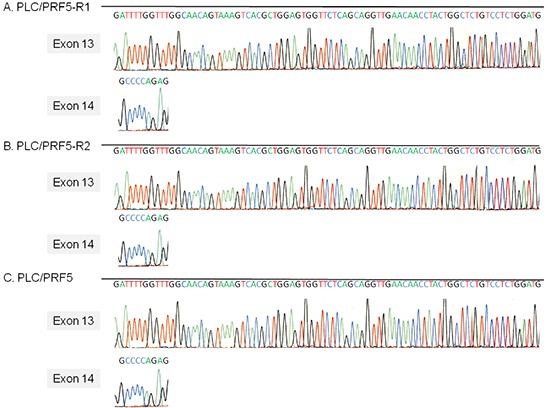
The activation segment sequence of the Raf1 gene in sorafenib-resistant cells The sequence of the activation segment of the Raf1 gene, which comprises exons 13 and 14, in PLC/PRF5, PLC/PRF5-R1 and PLC/PRF5-R2 cells was determined by direct sequencing.

Since sorafenib blocks the MAP kinase pathway mainly by inhibiting Raf1 kinase, we examined if there is a mutation in the activation segment of Raf1 kinase that involves binding with sorafenib (Figure 5). No mutation was detected in any of the sequences of exons 13 and 14 (activation segment) in the genomic DNA of PLC/PRF5-R1 and PLC/PRF5-R2 cells. These sequences were completely identical to those of parental cells. This suggests that sorafenib- resistance in PLC/PRF5-R1 and PLC/PRF5-R2 cells is not induced by mutation of the Raf1 gene.

## DISCUSSION

In this study, we established 2 sorafenib-resistant clones from the HCC cell line PLC/PRF5, and showed that themembrane transporter MRP3 was overexpressed in these clones. Moreover, knockdown of the MRP3 gene in these clones restored sensitivity to sorafenib. These data strongly suggest that MRP3 is a novel resistance factor for sorafenib. This is the first report showing a close association between sorafenib resistance and overexpression of a membrane transporter. Chen and associates reported that they established 2 sorafenib-resistant clones from theHCC cell line Huh7 [10]. Although they did not present IC50 values of the 2 sorafenib-resistant clones, the values appear to be about 10 μM based on the figure from their paper, and this represents 2-fold or less resistance relativeto parent cells. On the other hand, our 2resistant clones showed 1.8-fold and 4.6-fold resistance to sorafenib relative to parent cells. We believe that resistant cells with at least 3-fold or more resistance relative toparental cells shouldbe used for investigation of drug resistance. Moreover, Chen et al. demonstrated that the AKT/mTOR pathway was alternatively activated in their resistant clones. However, our resistant clones, with 1.8-fold and 4.6-fold resistance, did not show any enhancement of AKT/mTOR signals (Figure 2). The reason for this difference is unclear butmay be due to differences in the cell lines used. Moreover, although there no data are available on the expression of membrane transporters in their clones, it is possible that MRP3 is overexpressed in those cells. Since a majority of previous *in vitro* studies on sorafenib have used the HCC cell line PLC/PRF5 [25–27], we used these cells to establish sorafenib-resistance cell lines in the present study.

MRP3 is an ATP-binding cassette (ABC) transporter that is expressed in the basolateral membrane of hepatocytes and effluxes organic ions and glucuronide conjugates into the blood [28]. It is known that MRP3 is expressed in normal hepatocytes and it is reportedly sometimes overexpressed in HCC cells to various degrees [29]. Moreover, it has been reported that MRP3 is involved in resistance to etoposide and MTX in ovarian cancer and glioblastoma cells [30, 31]. Thus, it is plausible that HCC shows resistance to sorafenib depending on the extent of MRP3 expression. In fact, MRP3 was highly expressed in the HCC tissues that never responded to sorafenib treatment (3/5) but not in those that responded (0/4) by immunohistochemistry although the number of tissues (patients) examined was relatively small (Supplementary Figure S1).

Currently sorafenib is approved for the treatment of HCC and renal cell carcinoma [32, 33]. Since it is known that MRP3 is expressed not only in hepatocytes but also in renal cells [34], MRP3 may play an important role as a resistance factor in renal cell carcinoma.

Sorafenib undergoes oxidative metabolism mediated by cytochrome p450 3A4 primarily to sorafenib N-oxide [35]. Sorafenib and its oxidized metabolites undergo glucuronidation by UGT1A9 in hepatocytes, and is either excreted into bile and eliminated via feces or into the bloodstream and eliminated via urine [36]. However, it is currently unknown which transporters excrete glucuronide- sorafenib metabolites into bile or blood. MRP3 is a well-known transporter of glucuronide conjugates including etoposide and MTX into urine. Therefore, our data suggest that glucuronide conjugates of sorafenib metabolites are excreted by MRP3 into urine.

Sorafenib was originally developed to inhibit Raf1 kinase, and it exhibits a high antitumor activity by inhibiting RAF/MEK/ERK signal transduction. Since the RAF/MEK/ERK pathway is activated in a majority of HCC tissues [37], it is possible that a mutation occurs in the Raf1 gene, in particular in its activation segment, which is directly involved in binding to sorafenib in sorafenib-resistant cells. However, we did not find any mutations in thissegment of our sorafenib-resistant clones. Moreover, no mutations were detected in the ATP binding site of Raf1 kinase in these cells (Supplementary Figure S2). It has been reported that secondary mutations of the c-kit and EGFR genes confer acquired resistance to imatinib in GISTs and gefitinib in lung cancer respectively [22, 24]. These tumor cells have initialmutations as driver gene mutations, whereas HCC has few mutations in the Raf1 kinase gene. In addition, sorafenib inhibits not only Raf1 kinase but also B-Raf, PDGFR, VEGFR, and other tyrosine kinases. In this context, it is reasonable that genetic mutation is not involved in the resistance to sorafenib, although we cannot rule out thepossibility that there are mutations in other tyrosine kinases targeted by sorafenib in these resistant clones.

In conclusion, we established 2 sorafenib-resistant clones (PLC-PRF5-R1 and -R2) from the HCC cell line PLC/PRF5. These resistant clones showed overexpression of MRP3 but not other membrane transporters, and knockdown of MRP3 in the resistant clones restored the sensitivity to sorafenib. These results strongly suggest that MRP3 is a novel resistance factor for sorafenibin HCC cells.

## MATERIALS AND METHODS

### Cell culture and compounds

The representative human hepatoma cell line, PLC/PRF5, was purchased from American Tissue Culture Collection (ATCC). PLC/PRF5 cells were grown in Dulbecco's Modified Eagles Medium (DMEM, Invitrogen Sigma-Aldrich, St. Louis, MO) supplemented with 10% FBS, 2mM L-gultamine. Sorafenib (LKT Laboratory, St Paul, MN) was dissolved in 100% dimethyl sulfoxide (DMSO, Invitrogen Sigma-Aldrich) at 40 mM and stored at −20°C.

### Establishment of sorafenib-resistant cell line

PLC/PRF5 cells were continuously exposed to increasing concentrations of sorafenib over 24 months. Starting cell culture with 5.4 μM of sorafenib concentration (IC50 for PCL/PRF5 cells), the exposure dose was increased by 0.5 to 1 μM every month until reaching 3 times the IC50 inparent cells. Subsequently, cloning was performed by the limiting dilution method.

### Cell viability analysis

Cell viability was assessed by using the 3-(4,5-dimethylthiazol-2yl)-2,5- diphenyl-2H-tetrazolium bromide (MTT) assay. In brief, cells were seeded in 96-well plates (5000 cells/well) and incubated for 24 h at 37°C. Then, sorafenib was added to the wells at various concentrations, and the plates were incubated for another 72 h at 37°C. MTT dye solution (Sigma-Aldrich, St. Louis, MO) was added to the wells, and the plates were incubated at 37°C for 4 h, followed by addition of stop solution (isopropanol with 0.04 N HCl). Absorbance at 570 nm was determined on a (Bio-Rad, Hercules, CA) plate reader. IC50 values were determined by non-linear regression analysis. Values are given as mean ± standard deviation of 5 experiments.

### Western blot analysis

Cells were washed with phosphate buffered saline (PBS) and lysed in lysis buffer (50 mMol/L Tris pH 8.0, 150 mM NaCl, 0.01% SDS, 1% NP40, 0.5% Na-desoxycholate, 1 mM PMSF, 1 mM NaF, 1 mM Na_3_VO_4_) containing protease inhibitors (Sigma-Aldrich). Cell lysates were analyzed for protein content, resolved by sodium dodecylsulfate-polyamide gel electrophoresis (SDS-PAGE), and transferred to polyvinylidene fluoride (PVDF) membranes using a semi-dry transfer apparatus or wet transfer apparatus (Bio-Rad). Blots were blocked with 5% fat-free dry milk in Tris-buffered saline-Tween (TBS-T) buffer for 1 h and then incubated overnight with rabbit anti-phospho-Akt polyclonal antibody (Cell Signaling Technology, Tokyo, Japan), rabbit anti-Akt polyclonal antibody (Abcam, Cambridge, UK), rabbit anti-mTOR polyclonal antibody (Abcam), rabbit anti-phospho-mTOR (Abcam) monoclonal antibody, rabbit anti-ERK (Cell Signaling Technology) polyclonal antibody, mouse anti-phospho-ERK monoclonal antibody (Cell Signaling Technology), mouse anti-MDR1 monoclonal antibody (Santa Cruz Biotechnology, Santa Cruz, CA) monoclonal antibody, rabbit anti-MRP2 polyclonal antibody (Cell Signaling Technology), rabbit anti-MRP3 polyclonal antibody (Novus Biologicals, Littleton, CO), rabbit anti-MRP4 polyclonal antibody (Cell Signaling Technology), rabbit anti-BCRP polyclonal antibody (Sigma-Aldrich), rabbit anti-BSEP polyclonal antibody (Sigma-Aldrich), and rabbit anti-OCT1 polyclonal antibody (Abcam) as primary antibodies. The membranes were washed with TBS-T and incubated with secondary horseradish conjugated goat anti-mouse antibody (GE Healthcare UK Limited, Buckinghamshire, UK). The proteins were visualized by standard procedures including an ECL detection system (GE Healthcare UK Limited). To ensure equal protein loading, the same blot was developed for b-actin (Sigma-Aldrich) as a loading control.

### Transfection assay

The PCL/PRF5-R2 cells were transiently transfected with 30 nM of MRP3 specific small interfering RNA (siRNA) or random siRNA as a control using transfection reagent (Lipofectamine 2000, Invitrogen) according to the manufacturer's recommendations. After 24 h posttransfection, the cells were incubated with sorafenib for 48 h, and cell viability was determined by MTT assay. The knockdown of MRP3 gene was confirmed by real time PCR using Taqman gene expression assay of MRP3 (Hs00978473_m1, Applied Biosystems, Hitachi, Japan).

### Direct sequencing of Raf1 gene activation motif

Genomic DNA was isolated from cells using a DNeasy kit (QIAGEN, Hilden, Germany) following the manufacturer's instructions. The activation segment of Raf1 kinase encompassing exons 13 and 14 was amplified from DNA by PCR with the following primers: forward primer 5′-accagagtccttaacaagcattg-3′ and reverse primer 5′-gacagagccagtaggttgttcaa-3′ for exon 13, and the forward primer 5′-agatgtctgtgaggcctgtcata-3′ and reverse primer 5′-gatgccataggagtagacatccg-3′ for the exon 14. The PCR products were sequenced after purification using an ABI PRISM^®^3100 Genetic Analyzer (Applied Biosystems).

## SUPPLEMENTARY FIGURES


