# Supplementary material for: MRP3 as a novel resistance factor for sorafenib in hepatocellular carcinoma
Source: Oncotarget. 2016 Jan 12;7(6):7207–15. doi: 10.18632/oncotarget.6889 (PMC4872779; doi:10.18632/oncotarget.6889)
Supplement: Supplementary file 1 [file oncotarget-07-7207-s001.pdf]

SUPPLEMENTARY FIGURES

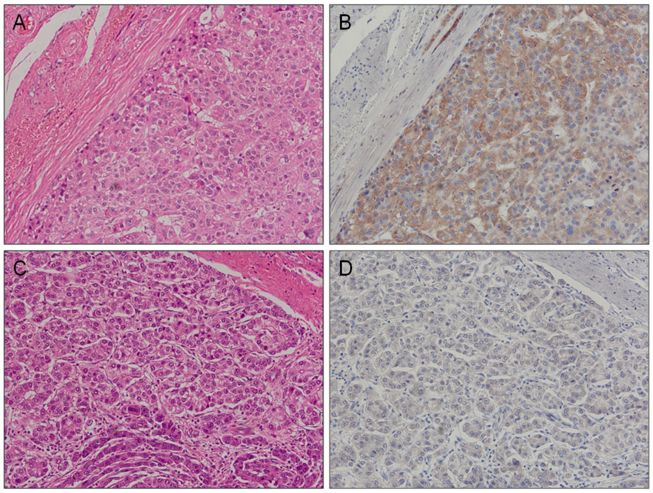

Supplementary Figure S1: Immunohistochemical analysis for MRP3 expression in hepatocellular carcinoma (HCC) tissues. Immunohistochemical staining was performed using a rabbit anti-MRP3 polyclonal antibody (Abcam) with labeled streptavidin biotin (LSAB) in HCC tissues from 9 patients (69.6 ± 6.8 years, M/F 7/2). Representative staining patterns of HCC tissues from a non-responder **A, B.** and responder **C, D.** to sorafenib are shown. Panels A and C, H&E staining (x200); Panels B and D, MRP3 staining of the corresponding HCC tissues (x200) respectively.

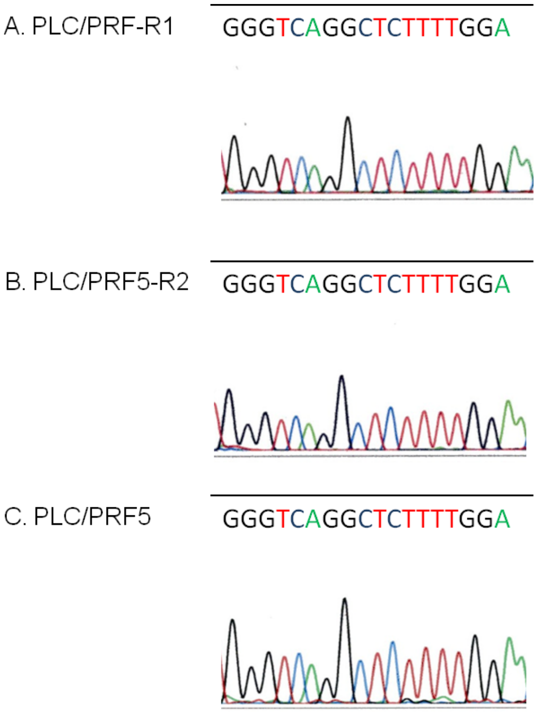

Supplementary Figure S2: The ATP binding site sequence of the Raf1 gene in sorafenib-resistant cells. The sequence of the ATP binding site of the Raf1 gene in PLC/PRF5, PLC/PRF5-R1 and PLC/PRF5-R2 cells was determined by direct sequencing.
